# Supplementary material for: Consumption of Sweet Beverages and Cancer Risk. A Systematic Review and Meta-Analysis of Observational Studies
Source: Nutrients. 2021 Feb 4;13(2):516. doi: 10.3390/nu13020516 (PMC7915548; doi:10.3390/nu13020516)
Supplement: Supplementary file 1 [file nutrients-13-00516-s001.pdf]

Table S1. Additional quantitative analysis for the association between sweet beverage consumption and cancer risk

Association	Subgroup	Subgroup category (n)	RR (95% CI)	I^2 (%)	Tau²	p between groups
SSB-Breast	Study type	Cohort (4)	1.19 (0.95-1.48)	0.0	0.0065	0.43
		Case-control (3)	1.09 (0.76-1.57)	0.0	0.0082	
	Country	US (5)	1.14 (0.93-1.40)	0.0	0.0100	0.98
		Med (2)	1.14 (0.43-3.00)	0.0	0.0038	
	Beverage intake category	High vs Low (4)	1.11 (0.89-1.39)	0.0	0.0083	0.61
		High vs None (3)	1.18 (0.80-1.76)	0.0	0.0085	
SSB-Breast Pre-M	Study type	Cohort (3)	1.60 (1.08-2.37)	0.0	0.0037	0.004
		Case-control (2)	1.08 (0.30-3.93)	0.0	0.0159	
	Country	US (3)	1.20 (0.61-2.36)	5.5	0.0377	0.24
		Med (2)	1.55 (0.22-10.9)	36.4	0.0302	
	Beverage intake category	High vs Low (4)	1.33 (0.88-2.00)	66.6	0.0371	0.45
		High vs None (1)	1.72 (0.92-3.25)	-	-	
SSB-Breast Post-M	Study type	Cohort (4)	1.16 (0.76-1.77)	12.4	0.0543	0.93
		Case-control (2)	1.21 (0.00-656.71)	86.6	0.3883	
	Country	US (3)	1.16 (0.35-3.86)	73.5	0.1775	0.90
		Other* (3)	1.20 (0.50-2.85)	41.6	0.0889	
	Beverage intake category	High vs Low (5)	1.21 (0.71-2.06)	63.8	0.1412	0.76
		High vs None (1)	1.11 (0.77-1.61)	-	-	
SB-Bladder	Overall Risk of Bias	Moderate (4)	1.27 (0.85-190)	25.3	0.0425	0.0001
		Serious (1)	4.73 (2.72-8.20)	-	-	
	Beverage intake category	High vs Low (1)	1.10 (070-1.71)	-	-	0.19
		High vs None (4)	1.86 (0.65-5.27)	86.7	0.3724	
SSB-Prostate	Country	US (2)	1.22 (0.80-1.85)	0.00	0.0008	0.049
		Other* (3)	1.13 (1.03-1.24)	0.00	0.0001	
	Beverage intake category	High vs Low (4)	1.20 (1.09-1.85)	0.00	0.0014	0.60
		High vs None (1)	1.13 (0.92-1.39)	-	-	
FJ-Prostate	Country	US (2)	1.03 (0.93-1.15)	0.00	<0.0001	0.27
		Other+ (2)	1.00 (0.75-1.34)	0.00	<0.0001	
	Beverage intake category	High vs Low (3)	1.03 (1.00-1.06)	0.00	<0.0001	0.69
		High vs None (1)	0.99 (0.80-1.22)	-	-	
SSB-Pancreatic	Study type	Cohort (4)	1.03 (0.90-1.18)	0.00	0.0020	0.15
		Case-control (2)	0.93 (0.50-1.75)	0.00	0.0003	
	Country	US (5)	1.03 (0.94-1.14)	0.00	0.0011	0.42
		EPIC (1)	0.90 (0.65-1.25)	-	-	
	Beverage intake category	High vs Low (2)	1.00 (0.24-4.27)	0.00	0.0081	0.98
		High vs None (4)	1.00 (0.90-1.13)	0.00	0.0006	

Supplementary Table S2. Continued.

ASB-Pancreatic	Study type	Cohort (3)	1.05 (0.92-1.21)	0.00	0.0003	0.03
		Case-control (2)	0.66 (0.37-1.17)	0.00	-	
	Country	US (4)	1.08 (0.67-1.74)	56.5	0.0689	0.77
		EPIC (1)	0.99 (0.61-1.60)	-	-	
	Beverage intake category	High vs Low (3)	0.93 (0.55-1.56)	0.00	0.0221	0.10
		High vs None (2)	1.27 (0.19-8.50)	50.9	0.0229	
SB-Pancreatic	Study type	Cohort (4)	1.42 (0.73-2.77)	80.8	0.1446	0.45
		Case-control (4)	1.29 (0.48-3.45)	43.1	0.1159	
	Country	US (4)	1.29 (0.71-2.34)	43.1	0.3404	0.99
		Other‡ (4)	1.29 (0.69-2.43)	72.7	0.3400	
	Beverage intake category	High vs Low (4)	1.16 (0.65-2.05)	43.1	0.1072	0.35
		High vs None (4)	1.47 (0.82-2.62)	71.8	0.0966	

None, non-consumer; p value ≤ 0.05 significant threshold; n: Number of studies in each subgroup; *France, Spain, Australia; + Sweden, France; ‡ 1 Asian and 3 European countries.

ASB: artificial sweetened beverages; EPIC: European Prospective Investigation into Cancer and Nutrition, includes: Denmark, France, Germany, Greece, Italy, Norway, Spain, Sweden, The Netherlands and United Kingdom. FJ: fruit juice; Med: Mediterranean countries; PI: prediction intervals; Post-M: post-menopausal; Pre-M: pre-menopausal; RR: risk ratio; CI: confidence interval; SB: sweetened beverages (includes both SSBs and ASBs); SSB: sugar sweetened beverages.

Table S2. Risk of bias in the included cohort studies according to ROBINS-E.

Study	Counf-ounding	Selection of participants into study	Classification of the exsposures	Departures from intended intervention	Missing data	Measu-rement of outcome	Selection of reported results	Overall bias
Larsson 2006	L	L	L	L	L	L	L	L
Nothlings 2007	L	L	L	L	L	L	L	L
Miles 2018	L	L	M	M	M	L	L	M
Pacheco 2019	L	L	M	M	M	L	M	M
Stepien 2014	L	L	L	M	L	L	L	L
Chazelas 2019	L	L	L	L	L	L	L	L
Navarrete-Muñoz 2016	L	L	L	L	L	L	L	L
Makarem 2018	L	L	M	L	NI	L	L	L
Zamora-Ros 2018	L	L	L	L	L	L	L	L
Lee 2006	M	L	L	M	M	L	L	M
Schernhammer 2012	L	L	L	L	L	L	L	L
Dubrow 2012	L	L	M	M	L	L	L	M
Inoue-Choi et al, 2013	L	L	M	L	NI	L	L	M
Schernhammer 2005	L	L	M	L	L	L	L	M
Mueller 2010	L	L	L	L	NI	L	L	M
Romano-Nanclares 2019	L	L	L	M	M	L	L	M
Hirvonen 2016	L	L	M	M	NI	L	L	M
Hodge 2018	M	L	M	M	M	L	L	M
Ren 2010	L	L	L	L	L	L	L	L
Drake 2012	L	L	L	L	L	L	L	L
Ellison 2000	M	L	L	M	NI	L	L	M
Nomura 2016	L	L	L	L	L	L	L	L
Ros 2011	L	L	L	L	L	L	L	L
McCullough 2014	L	L	L	M	M	L	L	M

Larsson 2016	L	L	L	L	L	L	L	L
Bao 2008	L	L	L	L	L	L	L	L
Bassett 2020	L	L	L	M	M	M	L	M

L, low risk of bias; M, moderate risk of bias; NI, not enough information for judgment.

Table S3. Risk of bias in the included Case-control studies according to NOS.

Study	Selection of cases and controls		Comparability of cases and controls		Ascertainment of exposure in cases and controls		Overall…	
	Stars (out of 4)	Risk of bias	Stars (out of 2)	Risk of bias	Stars (out of 3)	Risk of bias	Stars (out of 9)	Risk of bias
Leung 2016	***	M	*	M	*	M	*****	M
Hemelt 2010	***	M	**	L	*	M	******	M
Mahfouz 2014	***	M	NI	NI	*	M	****	M
Tayyem 2018	**	M	**	L	**	M	******	M
King 2013	***	M	**	L	*	M	******	M
Ibiebele 2008	***	M	**	L	*	M	******	M
Gallus 2011	**	M	**	L	*	M	*****	M
Theodorotau 2014	***	M	*	M	*	M	*****	M
Wang 2013	***	M	*	M	*	M	*****	M
Zvrko 2008	*	S	*	M	*	M	***	S
Murtaugh 2004	**	M		H	**	M	****	M
Song 2008	***	M	**	L	*	M	******	M
McLaughlin 1992	**	M	*	M	*	M	****	M
Chandran 2014	***	M	*	M	*	M	*****	M
Marzbani 2019	*	S	NI	NI	*	M	**	S
Potischman 2002	**	M	**	L	*	M	*****	M
Bener 2010	**	M	NI	NI	*	M	***	M
Mayne 2006	****	L	**	L	*	M	*******	L
Bruemmer 2007	**	M		S	*	M	***	S
De Stefani 2007	**	M	**	L	**	M	****	M
Radosavljević 2003	**	M		H	*	M	***	H
Turati 2015	**	M	*	M	*	M	****	M
Jain 1998	****	L		S	*	M	*****	S
Sharpe 2002	***	M	**	L	*	M	******	M
Hu 2009	***	M	**	L	*	M	******	M
Maclure and Willet, 1990	***	M	*	M	*	M	*****	M
Herrero 1991	**	M	*	M	*	M	****	M
Verreault 1989	***	M	**	L	*	M	******	M
Chan 2009	***	M	**	L	*	M	*****	M
Gold 1985	***	M	*	M	*	M	*****	M
Lyon 1992	**	M		S	*	M	***	S
Mack 1986	***	M	NI	NI	*	M	****	M
Lissowska 2003	**	M	*	M	*	M	****	M
Kreimer 2006	**	M	**	L	*	M	*****	M
Vincenti 2008	***	M	**	L	*	M	*****	M
Luqman 2014	**	M	NI	NI	*	M	***	S
Wu 1997	***	M	*	M	*	M	*****	M

L, low risk of bias; M, moderate risk of bias; S, serious risk of bias; NI, not enough information for judgment.

Breast

Study	TE	SE	High Total	Low Total	Weight (fixed)	Weight (random)	RR [95% CI]
Chandran et al_2014	-0.03	0.1378	1091	600	21.3%	20.3%	0.97 [0.74; 1.27]
Makarem et al_2018	0.04	0.2507	1045	717	6.4%	7.6%	1.04 [0.64; 1.70]
Potischman et al_2002	0.09	0.1603	1179	840	15.8%	16.2%	1.09 [0.80; 1.49]
Chazelas et al_2019	0.10	0.1195	59793	19931	28.4%	24.8%	1.10 [0.87; 1.39]
Chandran et al_2014	0.27	0.1864	420	1036	11.7%	12.7%	1.31 [0.91; 1.89]
Romanos-Nanclares et al_2019	0.31	0.3105	6650	4063	4.2%	5.1%	1.36 [0.74; 2.50]
Nomura et al_2016	0.34	0.1819	.	.	12.2%	13.2%	1.41 [0.99; 2.01]
**Total (fixed effect, 95% CI)**			.	.	**100.0%**	--	**1.13 [1.00; 1.28]**
**Total (random effects, 95% CI)**					--	**100.0%**	**1.14 [1.00; 1.29]**
**Prediction interval**							**[0.88; 1.47]**

Heterogeneity: Tau^2 = 0.0073; Chi^2 = 3.89, df = 6 (P = 0.69); I^2 = 0% [0%; 55%]

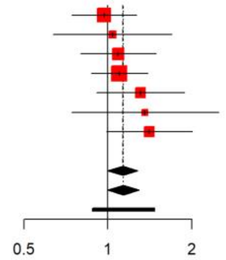

Breast (pre-menopausal)

Study	TE	SE	High Total	Low Total	Weight (fixed)	Weight (random)	RR [95% CI]
Chandran et al_2014	-0.05	0.2524	263	511	3.0%	15.5%	0.95 [0.58; 1.56]
Romanos-Nanclares et al_2019	0.15	0.2916	6180	3236	2.2%	12.8%	1.16 [0.66; 2.05]
Chandran et al_2014	0.16	0.2014	605	265	4.7%	20.2%	1.17 [0.79; 1.74]
Chazelas et al_2019	0.52	0.0465	51433	5468	88.2%	40.6%	1.68 [1.53; 1.84]
Nomura et al_2016	0.54	0.3231	419	292	1.8%	11.0%	1.72 [0.92; 3.25]
**Total (fixed effect, 95% CI)**			.	.	**100.0%**	--	**1.61 [1.48; 1.76]**
**Total (random effects, 95% CI)**					--	**100.0%**	**1.37 [1.00; 1.88]**
**Prediction interval**							**[0.68; 2.76]**

Heterogeneity: Tau^2 = 0.0358; Chi^2 = 9.03, df = 4 (P = 0.06); I^2 = 56% [0%; 84%]

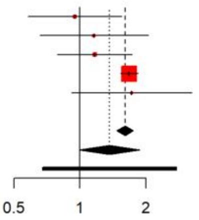

Breast (post-menopausal)

Study	TE	SE	High Total	Low Total	Weight (fixed)	Weight (random)	RR [95% CI]
Chandran et al_2014	-0.27	0.2007	486	335	15.5%	17.8%	0.76 [0.51; 1.13]
Chazelas et al_2019	-0.01	0.1678	9739	16043	22.2%	19.3%	0.99 [0.71; 1.38]
Hodge et al_2018	0.10	0.1362	16977	18616	33.6%	20.8%	1.11 [0.85; 1.45]
Nomura et al_2016	0.11	0.1889	.	.	17.5%	18.3%	1.11 [0.77; 1.61]
Chandran et al_2014	0.72	0.3026	157	525	6.8%	13.2%	2.05 [1.13; 3.71]
Romanos-Nanclares et al_2019	0.75	0.3760	1235	1610	4.4%	10.6%	2.12 [1.01; 4.43]
**Total (fixed effect, 95% CI)**			.	.	**100.0%**	--	**1.10 [0.94; 1.28]**
**Total (random effects, 95% CI)**					--	**100.0%**	**1.18 [0.79; 1.75]**
**Prediction interval**							**[0.43; 3.23]**

Heterogeneity: Tau^2 = 0.1080; Chi^2 = 11.07, df = 5 (P = 0.05); I^2 = 55% [0%; 82%]

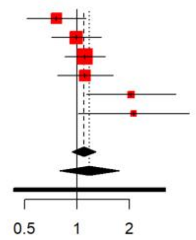

Colorectal

Study	TE	SE	High Total	Low Total	Weight (fixed)	Weight (random)	RR [95% CI]
Makarem et al_2018	-0.04	0.3245	2047	1136	5.9%	7.1%	0.96 [0.51; 1.81]
Chazelas et al_2019	0.01	0.2714	75943	25314	8.5%	10.1%	1.01 [0.59; 1.72]
Pacheco et al_2019	0.13	0.1469	58887	40911	28.9%	30.6%	1.14 [0.85; 1.52]
Hodge et al_2018	0.25	0.1051	16977	18616	56.6%	52.2%	1.28 [1.04; 1.57]
**Total (fixed effect, 95% CI)**			.	.	**100.0%**	--	**1.19 [1.02; 1.39]**
**Total (random effects, 95% CI)**					--	**100.0%**	**1.18 [0.99; 1.41]**
**Prediction interval**							**[0.82; 1.69]**

Heterogeneity: Tau^2 = 0.0039; Chi^2 = 1.37, df = 3 (P = 0.71); I^2 = 0% [0%; 66%]

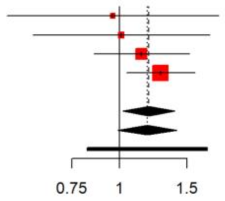

Pancreatic

Study	TE	SE	High Total	Low Total	Weight (fixed)	Weight (random)	RR [95% CI]
Chan et al_2009	-0.11	0.1972	982	1245	11.9%	12.2%	0.90 [0.61; 1.32]
Navarrete-Muñoz et al_2016	-0.11	0.1668	.	.	16.7%	16.8%	0.90 [0.65; 1.25]
Chan et al_2009	0.00	0.2802	793	1434	5.9%	6.2%	1.00 [0.58; 1.73]
Bao et al_2008	0.01	0.1355	122412	277202	25.3%	24.8%	1.01 [0.77; 1.32]
Nothlings et al_2007	0.07	0.1383	98264	63886	24.3%	23.9%	1.07 [0.82; 1.40]
Schernhammer et al_2005	0.12	0.1704	75598	62560	16.0%	16.1%	1.13 [0.81; 1.58]
**Total (fixed effect, 95% CI)**			.	.	**100.0%**	--	**1.01 [0.88; 1.15]**
**Total (random effects, 95% CI)**					--	**100.0%**	**1.01 [0.92; 1.11]**
**Prediction interval**							**[0.87; 1.17]**

Heterogeneity: Tau^2 = 0.0016; Chi^2 = 1.43, df = 5 (P = 0.92); I^2 = 0% [0%; 11%]

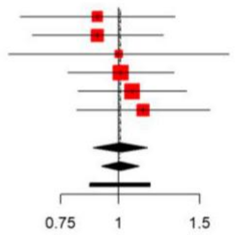

Prostate

Study	TE	SE	High Total	Low Total	Weight (fixed)	Weight (random)	RR [95% CI]
Hodge et al_2018	0.08	0.1668	16977	18616	9.4%	10.5%	1.08 [0.78; 1.50]
Drake et al_2012	0.12	0.1034	.	.	24.6%	25.7%	1.13 [0.92; 1.38]
Chazelas et al_2019	0.17	0.1859	16150	5383	7.6%	8.5%	1.19 [0.83; 1.71]
Miles et al_2018	0.19	0.0691	.	.	55.0%	51.3%	1.21 [1.06; 1.39]
Makarem et al_2018	0.32	0.2781	1188	464	3.4%	3.9%	1.38 [0.80; 2.38]
**Total (fixed effect, 95% CI)**			.	.	**100.0%**	--	**1.18 [1.07; 1.31]**
**Total (random effects, 95% CI)**					--	**100.0%**	**1.18 [1.10; 1.27]**
**Prediction interval**							**[1.03; 1.35]**

Heterogeneity: Tau^2 = 0.0012; Chi^2 = 0.91, df = 4 (P = 0.92); I^2 = 0% [0%; 8%]

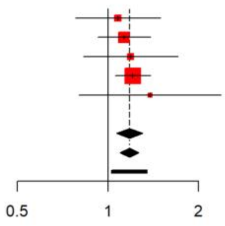

Figure S1. Forest plot showing pooled risk ratios with 95% CI for cancer risk, comparing the highest vs. lowest sugar-sweetened beverages (SSB) intake category.

Colorectal

Study	TE	SE	High Total	Low Total	Weight (fixed)	Weight (random)	RR [95% CI]
Tayyem et al_2018	0.33	0.3269	326	173	16.4%	32.4%	1.39 [0.73; 2.64]
Bener et al_2010	0.48	0.1532	95	333	74.8%	41.6%	1.62 [1.20; 2.19]
Mahfouz et al_2014	1.53	0.4482	118	332	8.7%	26.0%	4.60 [1.91; 11.07]
**Total (fixed effect, 95% CI)**			.	.	**100.0%**	--	**1.73 [1.33; 2.24]**
**Total (random effects, 95% CI)**					--	**100.0%**	**2.02 [0.45; 9.01]**
**Prediction interval**							**[0.00; 5753.09]**

Heterogeneity: $Tau^2 = 0.2711$; $Chi^2 = 5.39$, df = 2 (P = 0.07); $I^2 = 63\%$ [0%; 89%]

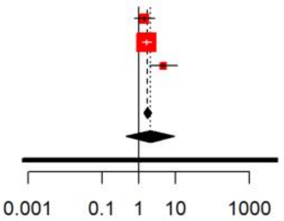

Pancreatic

Study	TE	SE	High Total	Low Total	Weight (fixed)	Weight (random)	RR [95% CI]
Navarrete-Muñoz et al_2016	-0.11	0.1427	288013	187176	27.3%	18.3%	0.90 [0.68; 1.19]
Chan et al_2009	0.00	0.1579	1768	459	22.3%	17.6%	1.00 [0.73; 1.36]
Gallus et al_2011	0.02	0.1768	284	693	17.8%	16.7%	1.02 [0.72; 1.44]
Lyon et al_1992	0.27	0.1988	271	224	14.1%	15.7%	1.31 [0.89; 1.93]
Mueller et al_2010	0.63	0.2684	14678	45846	7.7%	12.7%	1.87 [1.11; 3.16]
Larsson et al_2006	0.66	0.2497	36574	51223	8.9%	13.5%	1.93 [1.18; 3.15]
Mack et al_1986	0.96	0.5374	19	125	1.9%	5.5%	2.60 [0.91; 7.46]
Bao et al_2008	.	0.1112	.	.	0.0%	0.0%	
**Total (fixed effect, 95% CI)**			.	.	**100.0%**	--	**1.15 [0.99; 1.33]**
**Total (random effects, 95% CI)**					--	**100.0%**	**1.28 [0.95; 1.72]**
**Prediction interval**							**[0.56; 2.90]**

Heterogeneity: $Tau^2 = 0.0962$; $Chi^2 = 14.51$, df = 6 (P = 0.02); $I^2 = 59\%$ [5%; 82%]

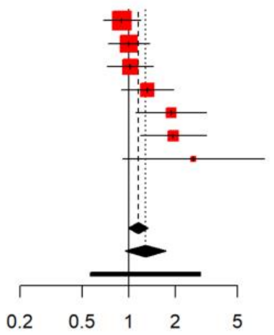

Bladder

Study	TE	SE	High Total	Low Total	Weight (fixed)	Weight (random)	RR [95% CI]
Turati et al_2015	0.04	0.1820	299	1056	21.7%	21.1%	1.04 [0.73; 1.49]
De Stefani et al_2007	0.10	0.2263	329	427	14.0%	20.1%	1.10 [0.71; 1.71]
Wang et al_2013	0.29	0.1229	934	473	47.6%	22.2%	1.34 [1.05; 1.71]
Hemelt et al_2010	0.70	0.3080	54	741	7.6%	18.0%	2.01 [1.10; 3.68]
Radosavljević et al_2003	1.55	0.2809	99	161	9.1%	18.7%	4.73 [2.73; 8.20]
**Total (fixed effect, 95% CI)**			.	.	**100.0%**	--	**1.43 [1.21; 1.69]**
**Total (random effects, 95% CI)**					--	**100.0%**	**1.66 [0.78; 3.56]**
**Prediction interval**							**[0.22; 12.38]**

Heterogeneity: $Tau^2 = 0.3226$; $Chi^2 = 24.04$, df = 4 (P < 0.01); $I^2 = 83\%$ [62%; 93%]

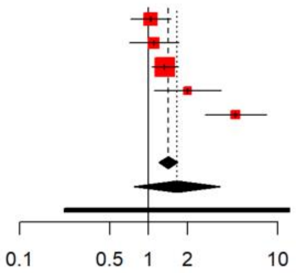

Prostate

Study	TE	SE	High Total	Low Total	Weight (fixed)	Weight (random)	RR [95% CI]
Jain et al_1998	-0.24	0.2020	449	804	35.6%	35.9%	0.79 [0.53; 1.17]
Sharpe et al_2002	0.00	0.1768	555	320	46.5%	42.0%	1.00 [0.71; 1.41]
Ellison et al_2000	0.25	0.2848	.	.	17.9%	22.1%	1.29 [0.74; 2.25]
**Total (fixed effect, 95% CI)**			.	.	**100.0%**	--	**0.96 [0.76; 1.22]**
**Total (random effects, 95% CI)**					--	**100.0%**	**0.97 [0.56; 1.70]**
**Prediction interval**							**[0.07; 12.67]**

Heterogeneity: $Tau^2 = 0.0241$; $Chi^2 = 2.06$, df = 2 (P = 0.36); $I^2 = 3\%$ [0%; 90%]

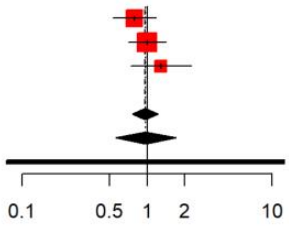

Renal cell

Study	TE	SE	High Total	Low Total	Weight (fixed)	Weight (random)	RR [95% CI]
Lee et al_2006	0.03	0.2462	.	.	21.5%	32.5%	1.03 [0.64; 1.67]
Hu et al_2009	0.23	0.1412	4494	1520	65.3%	40.0%	1.26 [0.96; 1.66]
Maclure and Willet_1990	0.96	0.3143	.	.	13.2%	27.6%	2.60 [1.40; 4.81]
**Total (fixed effect, 95% CI)**			.	.	**100.0%**	--	**1.33 [1.06; 1.66]**
**Total (random effects, 95% CI)**					--	**100.0%**	**1.44 [0.46; 4.50]**
**Prediction interval**							**[0.00; 604.16]**

Heterogeneity: $Tau^2 = 0.1559$; $Chi^2 = 5.77$, df = 2 (P = 0.06); $I^2 = 65\%$ [0%; 90%]

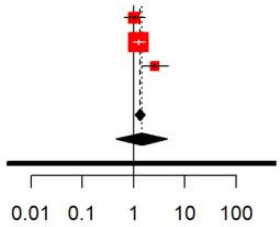

Figure S2. Forest plot showing pooled risk ratios with 95% CI for cancer risk, comparing the highest vs. lowest beverages with added sugar or non-sugar sweeteners (SB) intake category.

Breast

Study	TE	SE	High Total	Low Total	Weight (fixed)	Weight (random)	RR [95% CI]
Makarem et al_2018	0.03	0.2252	1189	623	16.2%	17.3%	1.03 [0.66; 1.60]
Chazelas et al_2019	0.12	0.1081	59794	19930	70.2%	67.9%	1.13 [0.91; 1.40]
Hirvonen et al_2006	0.25	0.2450	2139	2257	13.7%	14.7%	1.29 [0.80; 2.09]
**Total (fixed effect, 95% CI)**			.	.	**100.0%**	--	**1.13 [0.95; 1.35]**
**Total (random effects, 95% CI)**					--	**100.0%**	**1.13 [0.93; 1.38]**
**Prediction interval**							**[0.52; 2.46]**

Heterogeneity: $Tau^2 = 0.0017$; $Chi^2 = 0.46$, df = 2 (P = 0.79); $I^2 = 0\%$ [0%; 55%]

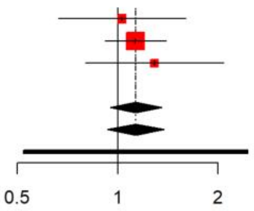

Colorectal

Study	TE	SE	High Total	Low Total	Weight (fixed)	Weight (random)	RR [95% CI]
Mahfouz et al_2014	-1.71	0.3534	100	350	18.1%	24.7%	0.18 [0.09; 0.36]
Tayyem et al_2018	0.07	0.4425	371	126	11.6%	23.1%	1.07 [0.45; 2.55]
Chazelas et al_2019	0.17	0.2161	75944	25313	48.5%	26.9%	1.19 [0.78; 1.82]
Makarem et al_2018	0.51	0.3229	2086	1097	21.7%	25.3%	1.66 [0.88; 3.13]
**Total (fixed effect, 95% CI)**			.	.	**100.0%**	--	**0.90 [0.67; 1.21]**
**Total (random effects, 95% CI)**					--	**100.0%**	**0.79 [0.16; 3.87]**
**Prediction interval**							**[0.01; 73.94]**

Heterogeneity: $Tau^2 = 0.8629$; $Chi^2 = 26.14$, df = 3 (P < 0.01); $I^2 = 89\%$ [73%; 95%]

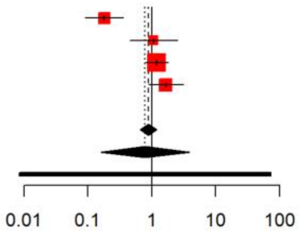

Prostate

Study	TE	SE	High Total	Low Total	Weight (fixed)	Weight (random)	RR [95% CI]
Drake et al_2012	-0.01	0.1045	.	.	1.3%	2.1%	0.99 [0.81; 1.22]
Makarem et al_2018	0.03	0.0123	1114	538	94.9%	91.8%	1.03 [1.01; 1.06]
Chazelas et al_2019	0.04	0.1594	16150	5383	0.6%	0.9%	1.04 [0.76; 1.42]
Miles et al_2018	0.07	0.0665	.	.	3.3%	5.2%	1.07 [0.94; 1.22]
**Total (fixed effect, 95% CI)**			.	.	**100.0%**	--	**1.03 [1.01; 1.06]**
**Total (random effects, 95% CI)**					--	**100.0%**	**1.03 [1.01; 1.05]**
**Prediction interval**							**[0.98; 1.09]**

Heterogeneity: $Tau^2 = 0.0001$; $Chi^2 = 0.47$, df = 3 (P = 0.93); $I^2 = 0\%$ [0%; 3%]

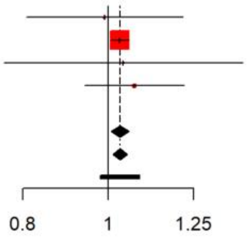

Figure S3. Forest plot showing pooled risk ratios with 95% CI for cancer risk, comparing the highest vs. lowest fruit juice (FJ) intake category.

Study	TE	SE	High Total	Low Total	Weight (fixed)	Weight (random)	RR [95% CI]
Gold et al_1985	-0.42	0.2933	.	.	6.4%	12.3%	0.66 [0.37; 1.17]
Navarrete-Muñoz et al_2016	-0.01	0.2460	.	.	9.1%	15.2%	0.99 [0.61; 1.60]
Schernhammer et al_2005	0.02	0.1309	74319	66839	32.2%	25.3%	1.02 [0.79; 1.32]
Bao et al_2008	0.10	0.1315	133533	268110	31.9%	25.3%	1.11 [0.86; 1.44]
Chan et al_2009	0.41	0.1649	901	1326	20.3%	21.9%	1.50 [1.09; 2.07]
**Total (fixed effect, 95% CI)**			.	.	**100.0%**	--	**1.10 [0.95; 1.27]**
**Total (random effects, 95% CI)**					--	**100.0%**	**1.07 [0.77; 1.48]**
**Prediction interval**							**[0.48; 2.36]**

Heterogeneity: $Tau^2 = 0.0480$; $Chi^2 = 7.09$, df = 4 (P = 0.13); $I^2 = 44\%$ [0%; 79%]

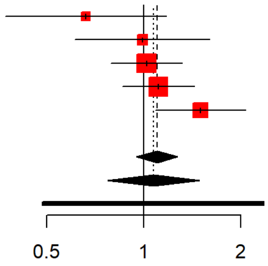

Figure S4. Forest plot showing pooled risk ratios with 95% CI for pancreatic cancer risk, comparing the highest vs. lowest artificial-sweetened beverages (ASB) intake category.
